# Happiness and physical activity levels of first year medical students studying in Cyprus: a cross-sectional survey

**DOI:** 10.1186/s12909-019-1790-9

**Published:** 2019-12-30

**Authors:** Joshua J. Fisher, Daphne Kaitelidou, George Samoutis

**Affiliations:** 1 St George’s University of London medical programme delivered by the University of Nicosia Medical School, 93 Agiou Nikolaou Street, 2408 Engomi, Nicosia Cyprus; 2 Open University of Cyprus, Health Management MSc Program, 33 Gianou Kranidioti Str., Latsia, Nicosia Cyprus; 3 Department of Nursing, National Kapodistrian of Athens, Athens, Greece

**Keywords:** Physical activity, Exercise, Medical students, Happiness, Depression

## Abstract

**Background:**

Levels of physical activity and happiness may impact the health and performance of future doctors. The specific relationship between physical activity and happiness among first year medical students is unclear. The purpose of this study was to investigate these variables and how they relate within first year, graduate entry Bachelor of Medicine, Bachelor of Surgery students studying in Cyprus.

**Methods:**

Self-administered questionnaires were provided for all first year medical students at the St. George’s University of London medical programme delivered by the University of Nicosia Medical School in Cyprus. Physical activity was assessed using the International Physical Activity Questionnaire Short Form and happiness was assessed using the Short Depression Happiness Scale. Surveys were completed by 79 of the 120 students (median age of 24 years). Happiness and continuous measures of physical activity amounts were investigated using spearman’s rank-order correlation. Mann-Whitney U Tests were used to make further comparisons between the physical activity levels across happy and depressed groups and gender, as well as to compare the levels of happiness reported by each gender.

**Results:**

High levels of physical activity were evident in 60.8% of students. Results suggested depression among 15.2% of students. A positive correlation was observed between happiness and amount of vigorous intensity physical activity among female students (*p* < 0.05), but not males. Happy females performed more vigorous physical activity than depressed females (*p* < 0.05). The total amount of physical activity performed, as well as level of happiness, did not significantly differ between genders.

**Conclusions:**

A relationship exists between physical activity and happiness among female first year medical students. The intensity of physical activity may play an important role within this group. There appears to be relatively high levels of physical activity and low levels of depression among male and female first year medical students studying in Cyprus. This study provides new knowledge regarding relationships between happiness and physical activity among first year medical students, and is also the first characterization of happiness and physical activity habits among students in Cyprus. This may help to inform future policies aimed at promoting health and wellness within student communities.

## Background

It is well established that physical activity and happiness can have substantial impacts on human health [1, 2]. Positive relationships between levels of physical activity and happiness have been previously reported in the literature [3–6], with previous research reporting that gender may play a role [6, 7]. However, the specific relationship between physical activity and happiness has not been established among first year medical students.

Medical students are often presented with demanding schedules and face many stressors. Research by Dahlin et al. suggests that the pressures of medical school may be worst during the first year of studies [8]. It is recognized that while many medical students meet or exceed minimum physical activity guidelines, many others have poor physical activity habits (e.g. [9–11]). Factors such as lack of energy due to academic activities and time limitations may contribute to previously observed low levels of physical activity [11]. Links between physical activity levels and academic performance have also been established among first year college students, with higher grades being associated with students’ engagement in regular physical activity [12]. Importantly, among medical students, low levels of physical activity are associated with burnout [9], decreased professional efficacy [13] and low health- related quality of life [9]. Since “doctors’ own physical activity practices influence their clinical attitudes towards physical activity” ([14], page 89), establishing optimal physical activity habits among medical students may have a critical influence on the health of their future patients.

Depression has been widely reported among medical students [15]. A recent meta- analysis by Puthran et al. reported “a global prevalence of depression amongst medical students of 28.0%” ([15], page 456). Previous research suggests that such levels of depression may be due to adjustment issues when beginning medical school, as well as various stressors throughout the duration of studies [15]. This has significant impact on students’ lives both during medical school [16] and continuing into their future professional lives, with high rates of physician suicide being reported in the literature [17].

The purpose of this study was to characterize and investigate relationships between the levels of physical activity and happiness among first year, graduate entry Bachelor of Medicine, Bachelor of surgery (MBBS) students studying in Cyprus. It was predicted that levels of physical activity and happiness would be positively correlated among first year medical students.

## Methods

Self-administered questionnaires were provided to graduate entry MBBS first year students studying at the St. George’s University of London medical programme delivered by the University of Nicosia Medical School in Cyprus. This represents Cyprus’ only MBBS programme. The questionnaire was made available to all first year students such that it coincided with a single compulsory problem based learning session already scheduled in their weekly timetable. All surveys were administered and completed on May 25, 2017, 27 days prior to the commencement of students’ end of year examinations. Students completed the survey on a voluntary basis. This study was approved by the Cyprus National Bioethics Committee.

The survey consisted of demographic information (i.e. age and gender) and two questionnaires; the International Physical Activity Questionnaire Short Form (IPAQ-SF) [18] and the Short Depression-Happiness Scale (SDHS) [19]. Previous research states that the IPAQ-SF has “acceptable reliability properties in Greek young adults” ([20], page 283) and a Cronbach’s alpha of 0.60 [21]. The SDHS has reported Cronbach’s alpha of 0.77 to 0.92 in the literature [19]. In both questionnaires, participants answered questions based on the 7 days preceding the completion of the survey. Incomplete surveys were removed from data analyses.

There were 120 first year medical students enrolled in the medical school at the time of this study. Surveys were administered to all 120 students. Seventy-nine students chose to complete the survey in full (*n* = 79; aged 21 to 45 years, median age of 24 years, mean age of 25.5 ± 4.51 years; 35 females, 44 males). Physical activity data were processed, weekly metabolic equivalent (MET)-minutes were calculated and participants were categorized into high, moderate and low physical activity level groups as per the IPAQ-SF recommended guidelines [22]. SDHS data were scored (negative questions scored in reverse) and a summative value for each participant was calculated on the recommended 0–18 scale [19]. Each participant was categorized into one of two groups (happy or depressed) on the basis of their total SDHS score. The happy group consisted of students with a score of ten or more, and the depressed group consisted of students with a total score of less than ten. This cut-off was chosen based on Joseph et al.’s suggestion that “a score below 10 on the SDHS might be taken as a cut-off point for mild but clinically relevant depression” ([19], page 10).

Statistical analyses were carried out in SPSS (IBM, Version 24). A type I error rate of 0.05 was used. Shapiro-Wilk Test of Normality revealed that data were not normally distributed, therefore nonparametric tests were used for subsequent statistical analyses. Spearman’s rank-order correlation was used to investigate relationships between SDHS score and each continuous measure of weekly physical activity amount (i.e. walking, moderate, vigorous and total). Mann-Whitney U Test was used to compare differences between each measure of weekly physical activity amount across happy and depressed groups and gender. Mann-Whitney U Test was also utilized to compare SDHS scores across genders. Cohen’s d values were calculated for Mann-Whitney U Test statistics to measure effect size [23] and interpreted with conventional definitions [24].

## Results

The majority of participants (60.8%) had a high level of physical activity (54.3% of females; 65.9% of males), whereas 32.9% demonstrated moderate levels (34.3% of females; 31.8% of males) and 6.3% had a low level of physical activity (11.4% of females; 2.3% of males) in the preceding week. The SDHS scores were less than ten in 15.2% of participants (Table 1).

**Table 1:** SDHS score and amounts of physical activity by gender and happiness

Participant		SDHS Score			Amount of Physical Activity (MET-minutes/week)											
					Vigorous Intensity			Moderate Intensity			Walking Intensity			Total Intensity		
		Median	Minimum - Maximum	Mean ± SD	Median	Minimum - Maximum	Mean ± SD	Median	Minimum - Maximum	Mean ± SD	Median	Minimum - Maximum	Mean ± SD	Median	Minimum - Maximum	Mean ± SD
Female	Happy (*N* = 30)	15,5	10–18	14.8 ± 2,23	1200	0–2880	1142.7 ± 999,29	240	0–4320	538.0 ± 860,38	536,25	0–4158	719.1 ± 865,94	2409,75	165–5973	2402.5 ± 1413,07
	Depressed (*N* = 5)	9	8–9	8.6 ± 0,55	0	0–800	160.0 ± 357,77	0	0–840	312.0 ± 429,32	693	0–1386	693.0 ± 693	693	0–3026	1165.0 ± 1349,63
	All Females (*N* = 35)	15	8–18	13.9 ± 3,02	960	0–2880	1002.3 ± 994,24	240	0–4320	505.7 ± 812,11	577,5	0–4158	715.4 ± 834,37	2106	0–5973	2225.7 ± 1452,73
Male	Happy (*N* = 37)	14	10–18	14.4 ± 1,85	1920	0–6720	1966.5 ± 1703	240	0–3360	532.4 ± 725,59	462	0–2772	707.3 ± 692,26	2826	495–10,212	3206.2 ± 2061,57
	Depressed (*N* = 7)	8	5–9	7.1 ± 1,77	960	0–3840	1200.0 ± 1292,44	300	0–2880	668.6 ± 1025,53	1155	33–4158	1301 ± 1475,66	2895	33–8106	3169.7 ± 2758
	All Males (*N* = 44)	14	5–18	13.3 ± 3,25	1440	0–6720	1844.5 ± 1655,78	240	0–3360	554.1 ± 768,15	462	0–4158	801.7 ± 867,95	2853	33–10,212	3200.4 ± 2149,37
Total	Happy	15	10 –	14.6 ±	1440	0 –	1597.6 ±	240	0 –	534.9 ±	462	0 –	712.6 ±	2655	165 –	2849.3 ±
	(*N* = 67)		18	2,02		6720	1480,23		4320	782,59		4158	768,71		10,212	1832,41
	Depressed	8	5 –	7.7 ±	400	0 –	766.7 ±	210	0 –	520.0 ±	924	0 –	1047.7 ±	1812	0 –	2334.4 ±
	(*N* = 12)		9	1,54		3840	1115,55		2880	821,22		4158	1208,5		8106	2424,25
	All	14	5 –	13.6 ±	1200	0 –	1471.4 ±	240	0 –	532.7 ±	495	0 –	763.5 ±	2586	0 –	2768.6 ±
	(N = 79)		18	3,15		6720	1455,88		4320	783,17		4158	848,89		10,212	1924,62

**Fig. 1:**
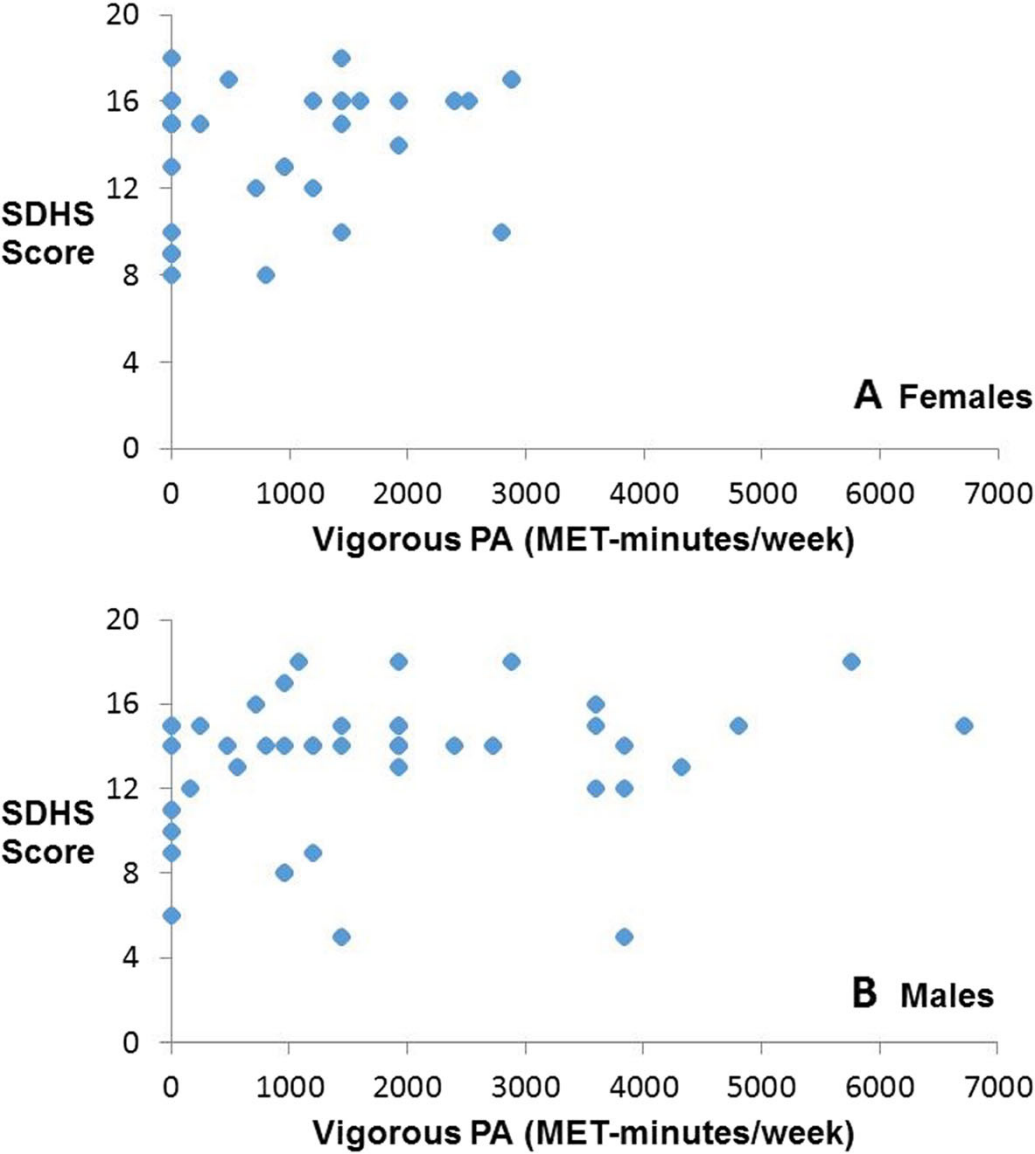
Plot of each participant’s amount of vigorous physical activity (MET-minutes/week) and SDHS scores. These variables were significantly and positively correlated (rs =0.382; *p* < 0.05) among females (**a**) but not among males (**b**)

Spearman’s rank-order correlation indicated a statistically significant, low positive correlation between SDHS score and amount of vigorous physical activity among females (Fig. 1a; rs(77) = 0.382, *p* = .024). However, the relationship between these variables was not statistically significant among men (Fig. 1b; rs(77) = 0.227, *p* = 0.139). No statistically significant correlations between SDHS score and moderate intensity (female: rs(77) = − 0.067, *p* = 0.703; male: rs(77) = − 0.219, *p* = 0.153), walking (female: rs(77) = − 0.077, *p* = 0.661; male: rs(77) = − 0.065, *p* = 0.676), or total physical activity (female: rs(77) = 0.234, *p* = 0.176; male: rs(77) = 0.109, *p* = 0.481) were observed.

Mann-Whitney U Tests revealed that the SDHS scores did not significantly differ between males and females, with a small effect size (U = 631, Z = − 1.384, *p* = 0.166, d = 0.312). Mann-Whitney U Tests also indicated that the female happy group performed significantly more vigorous physical activity in the preceding 7 days in comparison to the depressed group of females, with a medium effect size (U = 30, Z = − 2.182, *p* = 0.029, d = 0.768). This difference was not statistically significant between the male happy and depressed groups, with a small effect size (U = 93, Z = − 1.177, *p* = 0.239, d = 0.359). Amounts of moderate intensity, walking, or total physical activity did not differ significantly between the happy and depressed groups within either gender. Mann-Whitney U Tests revealed that the male group performed significantly more vigorous physical activity in the preceding 7 days in comparison to the female group, with a medium effect size (U = 543, Z = − 2.263, *p* = 0.024, d = 0.521). No differences of statistical significance were observed between the moderate intensity, walking, or total physical activity performed by females and males. Refer to Additional file 1 for further descriptive statistics.

## Discussion

The results of this study indicated that the amount of weekly vigorous intensity physical activity is positively correlated to happiness in female first year medical students studying in Cyprus. Previous findings based on data derived from the general populations of 15 European countries [6] indicated positive associations between both total amount of physical activity and happiness, and intensity of physical activity (e.g. vigorous physical activity) and happiness [6]. Similarly, data from a Scottish Health Survey revealed that both total amount and intensity of physical activity were associated with mental health benefits within the general Scottish population [4]. Positive associations between daily physical activity and happiness have also been reported among Chilean college students [5]. While our study did not identify any significant associations between total amount of physical activity and happiness, the findings did stress the important relationship that vigorous intensity physical activity may have with the happiness of medical students, particularly among females. This was supported by our finding that the depressed group of female students performed significantly less vigorous physical activity than their happy female peers, with a medium effect size noted, while differences in total amount of physical activity were not evident. Interestingly, similar findings were noted in research of the general European population [6], which reported associations between vigorous physical activity and happiness among females, but not males [6]. However, Richards et al. also observed positive associations between walking and happiness among participants, which was not evident in the current study. Differences between findings of Richards et al. and the current study may be due to participant demographics, since our study recruited only first year medical students, and Richards et al. focused on the general European population, including younger and older adult participants [6]. To our knowledge, the current study is the first report of a specific relationship between amount of physical activity and happiness among first year medical students, or among students studying in Cyprus.

The majority of students in the current study were physically active, with very few students (6.3%) falling within the low level of physical activity group. This was in contrast to previous studies which suggested that low levels of physical activity is more widespread among medical students, specifically a survey of over 4300 medical students in the United States reported that 37.3% did not meet minimal aerobic physical activity standards [9]. Our results illustrated that the total amount of physical activity performed by males and females did not differ significantly, and neither did their levels of happiness. However, in comparison to the female group, males completed a larger amount of vigorous physical activity, with a medium effect size noted. Interestingly, when considering the previously reported 28.0% “global prevalence of depression amongst medical students” ([15], page 460), the total number of depressed students in the current study (12/79) also seemed to be relatively low. The high level of physical activity reported in the current study may be influenced by campus layout and scheduling. In a typical school day, first year students have consecutive sessions scheduled at two different campuses, with a walking distance of approximately 1.2 km between them. This could account for a portion of students’ reported physical activity, which is likely encouraged by the walking/biking paths and local climate. Overall, our sample represents a novel group of interest within the literature, and it is possible that there are sociodemographic factors related to studying medicine in Cyprus which may have contributed to these findings, which provides an interesting topic for future research.

As the population of interest for the current study consisted of only 120 people (i.e. there were a total of only 120 first year MBBS students enrolled in Cypriot medical schools at the time of this study) and surveys were completed on a voluntary basis without incentives offered, a modest sample of 79 participants was achieved. This sample size, along with the possibility of response bias and self-selection bias are possible limitations of this study. The cross-sectional design of this study is also a limiting factor due to the inability to determine causation among relationships between variables.

## Conclusions

In conclusion, level of happiness was positively correlated to amount of vigorous physical activity in females, but not in males, within first year medical students studying in Cyprus. Neither level of happiness or total amount of exercise performed significantly differed between genders. To our knowledge, this is the first report of a specific relationship between levels of happiness and physical activity among first year medical students, and the first report of physical activity habits and happiness of students in Cyprus. As a practical implication, findings suggest that the inclusion of vigorous intensity physical activity may be of particular importance when encouraging physical activity among female students as it relates to level of happiness, as well as in the design of student wellness programs in general. Future research should strive to elucidate more detailed relationships between specific types of physical activity and happiness, as well as how these relationships may extend to specialty training and patient interactions. Furthermore, comparing these relationships and habits across students attending different medical schools could lead to the identification of factors related to the curriculum or geographic location which may influence the happiness and physical activity habits of future physicians.

## Supplementary information

**Additional file 1: Table S1.** SDHS score and amounts of physical activity by gender and happiness.

## Data Availability

The datasets used and/or analysed during the current study are available from the corresponding author on reasonable request.

## Abbreviations

IPAQ-SF: International Physical Activity Questionnaire Short Form
MBBS: Bachelor of Medicine, Bachelor of Surgery
MET: Metabolic equivalent
SDHS: Short Depression-Happiness Scale
